# Physical activity and metabolic syndrome in primary care patients in Spain

**DOI:** 10.1371/journal.pone.0317593

**Published:** 2025-01-24

**Authors:** Rafael Manuel Micó-Pérez, Natalia Hernández Segura, Vicente Martín-Sánchez, Alfonso Barquilla-García, Sonsoles M. Velilla-Zancada, José Polo-García, Miguel Ángel Prieto-Díaz, Vicente Pallares-Carratala, Antonio Segura-Fragoso, Leovigildo Ginel-Mendoza, Sergio Cinza-Sanjurjo

**Affiliations:** 1 Xàtiva–Ontinyent Department of Health, Specialist in Family and Community Medicine, Fontanars dels Alforins Health Center, Valencia, Spain; 2 Faculty of Health Sciences, Department of Biomedical Sciences, Area of Preventive Medicine and Public Health, Universidad de León, León, Spain; 3 Institute of Biomedicine (IBIOMED), The Research Group in Gene-Environment and Health Interactions (GIIGAS), Universidad de León, León, Spain; 4 Consortium for Biomedical Research in Epidemiology & Public Health (CIBER Epidemiología y Salud Pública-CIBERESP), Madrid, Spain; 5 Specialist in Family and Community Medicine, Trujillo Health Center, Cáceres, Spain; 6 Specialist in Family and Community Medicine, Joaquin Elizalde Health Center, Logroño, La Rioja, Spain; 7 Specialist in Family and Community Medicine, Casar de Cáceres Health Center, Cáceres, Spain; 8 Specialist in Family and Community Medicine, Vallobín-La Florida Health Center, Oviedo, Spain; 9 Department of Medicine, Jaume I University, Castellon, Spain; 10 Epidemiology Unit, Semergen Research Agency, Madrid, Spain; 11 Specialist in Family and Community Medicine, Ciudad Jardín Health Center, Málaga, Spain; 12 Specialist in Family and Community Medicine, Milladoiro Health Centre, Health Area of Santiago de Compostela, Health Research Institute of Santiago de Compostela (IDIS), Santiago de Compostela, Spain; 13 Networking Biomedical Research Centre-Cardiovascular Diseases (CIBERCV), Santiago de Compostela, Spain; Miami Cancer Institute, Baptist Health South Florida, UNITED STATES OF AMERICA

## Abstract

**Purpose:**

To determine the relationship between self-reported physical activity and the components of premorbid metabolic syndrome in patients treated in primary care according to sex.

**Methods:**

Cross-sectional descriptive study conducted on a sample of 2,359 patients without cardiovascular disease or diabetes, included in the cohort of the IBERICAN study. Using ANOVA models and adjusting for age, economic status, employment situation, level of education, adherence to a Mediterranean diet, tobacco use and alcohol consumption, we estimated the association of the variables blood pressure, triglycerides, HDL cholesterol, blood glucose and waist circumference with the self-reported level of physical activity (sedentary, moderate, high, very high). The analyses were performed stratifying by sex.

**Results:**

A total of 854 men and 1,505 women with no identified diseases were included. Women were more sedentary than men (*p*<0.004; OR = 1,35; IC95% = 1,10–1,65) and presented lower values in all the components of the metabolic syndrome, except for HDL-cholesterol, which was higher (p<0.001). The adjusted ANOVA model shows that diastolic blood pressure, triglycerides, fasting blood glucose, and waist circumference were significantly lower the higher the level of physical activity in both men and women (p<0.05).

**Conclusions:**

Patients served in primary care clinics without diabetes or cardiovascular disease and with high levels of physical activity showed better metabolic syndrome profiles. Given that women are more sedentary, gender approaches are needed in the promotion of physical activity to prevent metabolic syndrome and cardiovascular disease.

## 1. Introduction

Metabolic syndrome (MetS) is a global health concern, encompassing key cardiovascular risk factors such as diabetes, prediabetes, abdominal obesity, dyslipidemia, and high blood pressure. It is a significant precursor to cardiovascular diseases (CVD) and other chronic conditions [1,2]. Premorbid MetS is defined by the presence of three out of five diagnostic criteria: increased waist circumference, elevated blood pressure, high triglycerides, elevated blood glucose, and reduced high-density lipoprotein cholesterol (HDL-c) [3]. In Spain, the incidence of metabolic syndrome continues to with approximately more than 90,000 new cases diagnosed each year, partly due to sedentary lifestyles [4].

Physical inactivity is one of the main risk factors for mortality from non-communicable diseases, contributing to 830,000 deaths and 15.75 million disability-adjusted life years worldwide in 2019 [5]. It has been shown that people with an insufficient level of physical activity (PA) have a 20% to 30% higher risk of death compared to people who get a sufficient level of PA [6]. However, populations adhering to healthier lifestyles exhibit higher life expectancy and quality of life, with reduced morbidity and mortality [7]. Despite this, recent surveys indicate a decline in PA levels in Western societies compared to previous generations with a prevalence of sedentary behavior of 25.7% in Spain [1,8,9]. The World Health Organization (WHO) recommends at least 150 minutes of moderate-intensity PA or 75 minutes of vigorous-intensity PA per week for adults [10,11]. Non-compliance with these recommendations is linked to increased risks of coronary heart disease, cancer, diabetes, anxiety, depression, cognitive impairment, and reduced life expectancy [12,13].

Primary Care (PC) plays a crucial role in disease prevention and health promotion by encouraging healthy lifestyles [14]. The potential impact of promoting PA from PC is substantial, with estimates suggesting that 20% of deaths could be prevented if individuals met minimum PA recommendations [15,16]. Despite this, the incorporation of PA into MetS prevention strategies remains underutilized [1].

The prevalence of MetS increases with age, is higher in men than women, and varies by race and ethnicity. There is an inverse relationship between PA and MetS, more pronounced in men, and it is known that a physically active lifestyle can prevent or delay the onset of MetS in young adults [17–20]. Recent evidence underscores the importance of a sex-specific approach to promote PA more effectively and to deepen our understanding of sex-based differences in the development and impact of MetS and cardiovascular disease (CVD). Such an approach could lead to improved health outcomes and more tailored healthcare strategies [21,22].

In the IBERICAN study (Identification of the Spanish Population at Cardiovascular and Renal Risk), carried out on Spanish adult population served in PC to identify the Spanish adult population at cardiovascular and renal risk, one of the secondary objectives was to determine the impact of healthy lifestyles, specifically PA, on CVD [23,24]. In this study, we aim to study the effect of self-reported PA on the components of MetS and, consequently, on the prevention of CVD, according to sex. We analyzed this relationship in patients without diabetes mellitus (DM) or CVD included in the cohort of the IBERICAN study, differentiating by sex and other sociodemographic factors.

## 2. Material and methods

### 2.1. Study design

The IBERICAN study is an epidemiological, multicenter, observational, prospective study that is being carried out in PC in Spain in patients of the National Healthcare System, and whose design and characterization of the population have already been published [25]. Fieldwork for this sub-study was conducted between June 2014 and December 2018. A total of 519 family physicians participated in the study, who used consecutive sampling to select at least 10 patients who met the following inclusion criteria: (1) be between 18 and 85 years old, (2) be a user of the Spanish National Health System and attend a PC centre and (3) be resident in Spain within the last 5 years.

The study was classified by the Spanish Agency for Medicines and Health Products (AEMPS) as a Non-Post-Authorization Observational Study (Non-PAS) on January 23, 2013. It was approved by the Clinical Research Ethics Committee (CREC) of Hospital Clínico San Carlos in Madrid on February 21, 2013 (CP IBERICAN-CI13/047-E) and is registered at https://clinicaltrials.gov under number NCT02261441. The results provided in this paper correspond to a cross-sectional analysis of the subjects included in the study up to December 15, 2018.

### 2.2. Data collection

The subjects included in the study underwent a conventional examination of clinical and analytical parameters according to standard clinical practice. Analytical determinations were considered valid if they had been performed up to six months before the patient’s inclusion in the study or if they were conducted at the time of inclusion.

The data collection process from the case report forms (CRF) was carried out using remote capture: e-clinical. This process refines the data entry techniques by filtering CRF variables, thereby reducing errors and reducing the time between data collection processes and the publication of study results. The researcher accessed a public Uniform Resource Locator (URL) on the Internet, which required him to identify himself as a member of the research community of SEMERGEN (Spanish Society of Primary Care Physicians) with a username and password. Once validated, he accessed an implemented data collection system. All collected information can be consulted in the study by Cinza Sanjurjo et al. [25].

### 2.3. Study variables

The exposure variable was categorized into four categories (sedentary, moderate, high, and very high) based on the PA practice reported by the patients in the baseline questionnaire. Those who reported not exercising at all were classified as sedentary. People who actively exercised between 30 and 60 minutes a day were classified into the moderate group. Those who actively exercised for at least 60 minutes a day were assigned to the high moderate group. Finally, the subjects who indicated that they regularly practiced sports were classified into the very high group.

Systolic blood pressure (SBP) and diastolic blood pressure (DBP) measured in mmHg, as well as HDL cholesterol, triglyceride and fasting blood glucose concentrations in mg/dl and waist circumference in cm were used as outcome variables.

### 2.4. Statistical analysis

First, a descriptive study of the sample of 2359 patients without CVD and DM was carried out (S1 Fig). To analyze differences between men and women, the chi-squared test was used for qualitative variables, and the Wilcoxon test for quantitative variables, due to the lack of normality in the data. To analyze differences between sexes based on the level of physical activity, a logistic regression was conducted.

Next, two models of ANOVA analysis of variance were created: model I, adjusting for age, socioeconomic status, employment situation and level of education; and model II, adjusting for age, socioeconomic status, employment situation, level of education, adherence to a Mediterranean diet, tobacco use, family history of cardiovascular event, and alcohol consumption. These two models were used to evaluate whether there were differences based on the level of PA (sedentary, moderate, high and very high) in SBP, DBP, as well as in triglycerides, HDL cholesterol, blood glucose and waist circumference stratified by sex. Statistically significant interactions were explored and nominal p values and their 95% confidence intervals (95% CI) were reported. *P*-values < 0.05 were considered statistically significant. The Stata version 16 statistical software was used, as well as version R 3.6 for graphs and statistical calculations.

## 3. Results

The study included 2359 patients, 854 men and 1505 women, whose characteristics are presented in Table 1. The proportion of women who did not do PA was 25.65% compared to 20.37% of men (*p*<0.004; OR = 1.35; IC95% = 1.10–1.65). On the other hand, 20.02% of men had a very high level of PA compared to 8,97% of women (*p*<0.001; OR = 2.54; IC95% = 1.99–3.24). No significant differences were found between men and women in employment situation, level of education, adherence to a Mediterranean diet and family history of cardiovascular events (*p*>0.05). On the contrary, in addition to the level of PA, statistically significant differences were found according to sex in economic level, tobacco use, alcohol consumption, BMI, age, SBP, DBP, triglycerides, HDL cholesterol, blood glucose levels and waist circumference (p<0.05).

**Table 1:** Clinical, metabolic, and body composition characteristics of the participants.

Variable	Category	Men	Women	*P value*
		(n = 854)	(n = 1505)	
**Physical activity** (%)	None (sedentary)	174 (20.37)	386 (25.65)	**<0.001**
	Moderate	304 (35.60)	636 (42.26)	
	High	205 (24.00)	348 (23.12)	
	Very high	171 (20.02)	135 (8.97)	
**Economic level** (%)	Less than €18,000	259 (30.33)	541 (35.95)	**0.006**
	Annual income from €18,000	595 (69.67)	964 (64.05)	
**Employment situation** (%)	Work/Unemployed	655 (77.61)	1130 (75.69)	0.294
	Retired/ Student/ Housework	189 (22.39)	363 (24.31)	
**Tobacco use** (%)	Non-smoker	369 (43.77)	898 (60.35)	**<0.001**
	Ex-smoker	245 (29.06)	294 (19.76)	
	Current	229 (27.16)	296 (19.89)	
**Level of education** (%)	No education/Primary education	412 (48.24)	693 (46.05)	0.304
	Higher than primary	442 (51.76)	812 (53.95)	
**Alcohol consumption** (%)	No	699 (82.72)	1416 (94.97)	**<0.001**
	Yes	146 (17.28)	75 (5.03)	
**BMI (Kg/m**^**2**^**)** (%)	<25	292 (34.19)	736 (48.9)	**<0.001**
	25–29.9	384 (44.96)	488 (32.43)	
	≥30	178 (20.84)	281 (18.67)	
**Adherence to a Mediterranean diet** (%)	Very low	215 (25.53)	365 (24.60)	0.481
	Low	231 (27.43)	429 (28.91)	
	Moderate	184 (21.85)	350 (23.58)	
	High	212 (25.18)	340 (22.91)	
**Family history of cardiovascular events** (%)	No	728 (91.69)	1242 (89.10)	0.052
	Yes	66 (8.31)	152 (10.90)	
**Age (years)**	Mean (SD)	47.46 (15.19)	46.03 (13.83)	0.04*
**Systolic blood pressure (mmHg)**	Mean (SD)	125.42 (13.36)	118.35 (14.50)	<0.001*
**Diastolic blood pressure (mmHg)**	Mean (SD)	75.96 (9.71)	72.78 (9.92)	<0.001*
**Triglycerides (mg/dl)**	Mean (SD)	114.42 (64.87)	93.26 (56.28)	<0.001*
**HDL cholesterol (mg/dl)**	Mean (SD)	51.45 (13.67)	61.75 (15.30)	<0.001*
**Blood glucose levels (mg/dl)**	Mean (SD)	92.40 (13.67)	88,20 (11,64)	<0.001*
**Waist circumference (mmHg)**	Mean (SD)	94.94 (12.83)	86.25 (14.08)	<0.001*

BMI: Body mass index, SD: Standard desviation.

* Test de Wilcoxon.

BMI: Body mass index.

**Fig 1:**
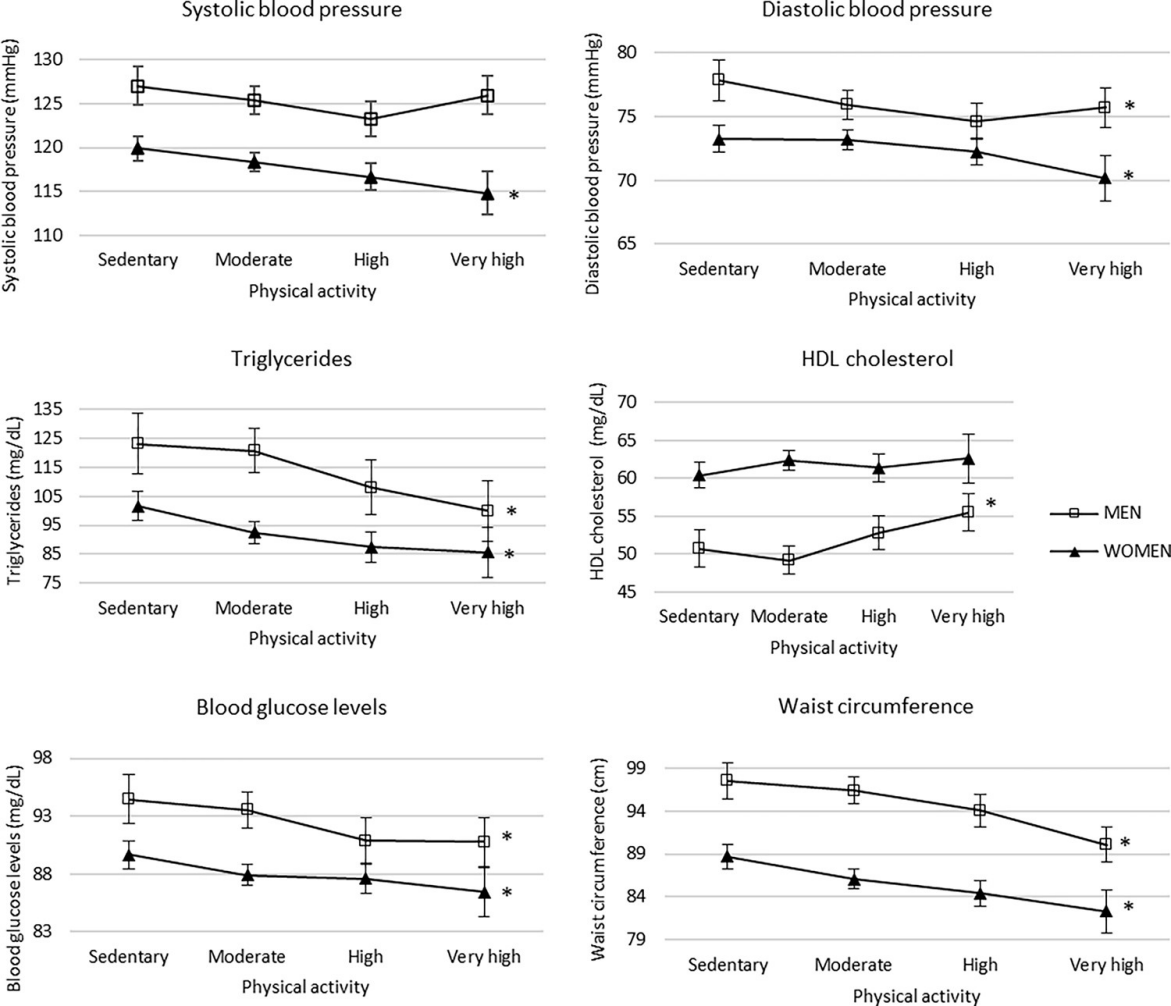
Graphical representation of the adjusted ANOVA results. Model adjusted for age, socioeconomic status, employment situation, level of education, physical activity, Mediterranean diet, tobacco use, alcohol consumption, and family history of cardiovascular event. * *p*<0.05.

Table 2 and Fig 1 show that in women, a statistically significant trend towards lower SBP and DBP, lower triglyceride and glucose levels, as well as lower waist circumference, was observed with higher self-reported physical activity levels. Specifically, women with very high levels of physical activity had an average SBP of 114.78 mmHg and an average DBP of 70.18 mmHg, compared to 119.88 mmHg (*p* = 0.001) and 73.25 mmHg (*p* = 0.017), respectively, in sedentary women. Additionally, triglyceride and glucose levels in women with very high physical activity were 85.58 mg/dL and 86.41 mg/dL, respectively, compared to 101.61 mg/dL (*p* < 0.001) and 89.66 mg/dL (*p* = 0.027) in sedentary women. Waist circumference was also lower in athletic women (82.31 cm) compared to sedentary women (88.68 cm, *p* < 0.001).

**Table 2:** Results of the analysis of variance models on the effect of physical activity on clinical characteristics.

**Physical Activity Levels**	**Model I** *				**Model II** **			
	**Men**		**Women**		**Men**		**Women**	
	**Systolic blood pressure**							
	**mmHg (95% CI)**	***p-value***	**mmHg (95% CI)**	***p-value***	**mmHg (95% CI)**	***p-value***	**mmHg (95% CI)**	***p-value***
**Sedentary**	127.49 (125.43–129.56)	**0.022**	119.86 (118.49–121.24)	**<0.001**	127.05 (124.90–129.21)	0.073	119.88 (118.46–121.31)	**0.001**
**Moderate**	125.34 (123.82–126.87)		118.96 (117.90–120.01)		125.44 (123.85–127.02)		118.36 (117.26–119.45)	
**High**	123.13 (121.27–124.99)		116.56 (115.14–117.99)		123.27 (121.30–125.24)		116.66 (115.19–118.14)	
**Very high**	125.81 (123.74–127.88)		115.38 (113.02–117.74)		125.99 (123.85–128.13)		114.78 (112.34–117.21)	
** **	**Diastolic blood pressure**							
	**mmHg (95% CI)**	***p-value***	**mmHg (95% CI)**	***p-value***	**mmHg (95% CI)**	***p-value***	**mmHg (95% CI)**	***p-value***
**Sedentary**	78.25 (76.74–79.76)	**0.004**	73.22 (72.22–74.21)	**0.010**	77.84 (76.28–79.41)	**0.033**	73.25 (72.21–74.29)	**0.017**
**Moderate**	75.85 (74.74–76.97)		73.32 (72.56–74.08)		75.94 (74.79–77.10)		73.17 (72.37–73.97)	
**High**	74.44 (73.08–75.81)		72.05 (71.02–73.08)		74.61 (73.18–76.04)		72.25 (71.18–73.33)	
**Very high**	75.60 (74.09–77.11)		70.40 (68.70–72.11)		75.71 (74.15–77.27)		70.18 (68.41–71.96)	
** **	**Triglycerides**							
	**mg/dL (95% CI)**	***p-value***	**mg/dL (95% CI)**	***p-value***	**mg/dL (95% CI)**	***p-value***	**mg/dL (95% CI)**	***p-value***
**Sedentary**	126.12 (115.73–136.51)	**<0.001**	100.38 (95.54–105.21)	**<0.001**	123.23 (112.65–133.8)	**0.005**	101.61 (96.5–106.71)	**<0.001**
**Moderate**	121.50 (113.83–129.16)		92.75 (89.05–96.45)		120.76 (112.98–128.54)		92.40 (88.48–96.32)	
**High**	109.05 (99.69–118.4)		86.40 (81.40–91.40)		108.11 (98.44–117.78)		87.46 (82.17–92.75)	
**Very high**	98.67 (88.27–109.06)		83.38 (75.09–91.68)		99.94 (89.4–110.48)		85.58 (76.86–94.31)	
** **	**HDL cholesterol**							
	**mg/dL (95% CI)**	***p-value***	**mg/dL (95% CI)**	***p-value***	**mg/dL (95% CI)**	***p-value***	**mg/dL (95% CI)**	***p-value***
**Sedentary**	50.80 (48.50–53.10)	**<0.001**	60.28 (58.63–61.93)	0.184	50.73 (48.30–53.16)	**<0.001**	60.40 (58.67–62.12)	0.302
**Moderate**	49.12 (47.42–50.82)		62.34 (61.09–63.59)		49.18 (47.38–50.97)		62.39 (61.08–63.70)	
**High**	52.40 (50.33–54.47)		61.88 (60.14–63.62)		52.78 (50.56–55.01)		61.39 (59.56–63.21)	
**Very high**	55.29 (52.92–57.65)		63.36 (60.30–66.41)		55.48 (52.99–57.97)		62.61 (59.43–65.80)	
** **	**Fasting blood glucose**							
	**mg/dL (95% CI)**	***p-value***	**mg/dL (95% CI)**	***p-value***	**mg/dL (95% CI)**	***p-value***	**mg/dL (95% CI)**	***p-value***
**Sedentary**	94.65 (92.59–96.71)	**0.004**	89.46 (88.29–90.64)	0.057	94.45 (92.32–96.58)	**0.025**	89.66 (88.44–90.88)	**0.027**
**Moderate**	93.48 (91.96–95.00)		87.96 (87.06–88.86)		93.53 (91.97–95.1)		87.89 (86.96–88.83)	
**High**	90.91 (89.06–92.78)		87.70 (86.49–88.92)		90.87 (88.93–92.82)		87.58 (86.31–88.84)	
**Very high**	90.04 (89.98–92.10)		86.57 (84.55–88.59)		90.75 (88.63–92.87)		86.41 (84.33–88.5)	
** **	**Waist circumference**							
	**cm (95% CI)**	***p-value***	**cm (95% CI)**	***p-value***	**cm (95% CI)**	***p-value***	**cm (95% CI)**	***p-value***
**Sedentary**	98.26 (96.30–100.22)	**<0.001**	88.97 (87.59–90.36)	**<0.001**	97.51 (95.40–99.62)	**<0.001**	88.68 (87.25–90.12)	**<0.001**
**Moderate**	96.46 (95.02–97.90)		86.31 (85.26–87.37)		96.34 (94.82–97.87)		86.08 (84.99–87.18)	
**High**	94.03 (92.26–94.80)		84.61 (83.18–86.05)		94.06 (92.15–95.97)		84.42 (82.94–85.91)	
**Very high**	89.86 (87.91–91.81)		82.44 (80.07–84.82)		90.07 (88.00–92.14)		82.31 (79.86–84.76)	

*Model adjusted for age, socioeconomic status, employment situation and level of education.

** Model adjusted for age, socioeconomic status, employment situation, level of education, physical activity, Mediterranean diet, tobacco use, alcohol consumption, and family history of cardiovascular event.

In men, a statistically significant trend towards lower DBP, triglyceride, and glucose levels, as well as lower waist circumference and higher HDL cholesterol level, was observed with higher self-reported physical activity levels. Men with very high levels of physical activity showed an average DBP of 75.71 mmHg, triglyceride levels of 99.94 mg/dL, and glucose levels of 90.75 mg/dL, compared to 77.84 mmHg (*p* = 0.033), 123.23 mg/dL (*p* = 0.005), and 94.45 mg/dL (*p* = 0.025), respectively, in sedentary men. Waist circumference in men with very high physical activity was 90.07 cm, while in sedentary men it was 97.51 cm (*p* < 0.001). Additionally, HDL cholesterol levels were higher in men with very high physical activity (55.48 mg/dL) compared to sedentary men (50.73 mg/dL, *p* < 0.001).

## 4. Discussion

Our study showed that patients, both men and women, who report more PA have better profiles in the components of MetS, such as better blood pressure, blood glucose, waist circumference and triglyceride or HDL-cholesterol levels.

Physical inactivity represents a significant and modifiable risk factor that is more prevalent and severe in the female population globally for all age groups. The gender gap in PA begins early in life and leads to considerable short- and long-term adverse effects on health outcomes, especially CV health [26]. Our study also shows that women are more sedentary than men (OR = 1.35; 95% CI = 1.10–1.65), so they could particularly benefit from increasing their PA levels.

As regards PA and sport, male subjects seem to be more active than female subjects [26]. The results of several studies show that men’s attitudes are more positive than women’s, with significant differences [27]. In the 2022 Survey of Sports Habits in Spain [28], sports practice continues to be higher among men, regardless of frequency, standing at 63.1% and 51.8% respectively in annual terms. The gap, 11.3 percentage points, is lower than that recorded in 2015, 12.3 percentage points, and similar to that recorded in our study (11.1%) whose data were collected between 2014 and 2018.

Regular PA reduces sympathetic activity and plasma catecholamine concentration at rest, and modifies renal homeostasis with a decrease in vascular resistance, contributing to a reduction in blood pressure [29]. High blood pressure is the main independent cardiovascular risk factor [30]. PA reduces SBP by an average of 6.9 mmHg in people with hypertension [31]. Cordero *et al*. [29] speak of an average reduction of 6–7 mmHg in SBP and DBP in hypertensive patients compared to 3 mmHg in normotensive patients. Cornelissen *et al*. [32], in a systematic review with healthy adults, found a significant reduction in DBP in patients who received a training program, regardless of the type of training; but they found no reduction in SBP in patients who received combined strength and endurance training. In terms of sex, they found that men with a training program achieved more than twice this reduction in SBP and DBP than women. Our results also show differences in terms of sex, but in this case women show a greater reduction in blood pressure than men.

Regarding the lipid profile, we also found evidence that PA improves it. The literature consulted mainly shows an increase in HDL values and a decrease in triglycerides in patients who practice PA [33–37]. In women, there is greater lipolytic activity in response to exercise [38]. Our results are in line with these studies, showing a statistically significant decrease in triglyceride levels in both sexes, but only a statistically significant increase in HDL cholesterol in the case of men. Therefore, it would be advisable to continue this line of research to verify whether the PA manages to improve the lipid profile in healthy individuals.

One of the direct effects of PA is to decrease insulin resistance, since muscle tissue increases glucose uptake. Regular PA decreases the risk of type 2 diabetes mellitus (T2DM) and observational studies suggest that in patients with T2DM, women may require greater frequency and intensity of PA than men to reduce CV events [39]. In this sense, aerobic PA leads to an increase in the biological efficacy of insulin, and it has been reported that, even after training, sensitivity and the number of insulin receptors increase by 36% [40]. In our study on healthy patients, a reduction in blood glucose levels was observed, and unlike other studies consulted, our data showed significant differences. Significant differences between men and women were only observed in the lowest PA levels, while other studies reported no difference between sexes [36,41].

On the other hand, the results obtained show a reduction in waist circumference as the PA level increases, in this case more marked in men than in women, in line with the existing literature [36]. The regular practice of PA is effective in the prevention of overweight and in the treatment of obesity-related comorbidities [42], and thus PA improves CVR due to body weight regulation.

Among the possible limitations of our study, it should be noted that the collection of data on PA levels comes from a self-reported questionnaire in which the type and intensity of activity performed are not specified, although according to Obling et al. [43] the use of a single item to measure PA levels is a cost-effective strategy. Another limitation of the database is the lack of information on aspects related to sleep quality, emotional health, environmental pollution or maximum oxygen consumption (VO2max). It is also worth noting that the data were collected between 2014 and 2018. However, a database with 2,359 patients from all over Spain, as well as socioeconomic variables that provide robustness to the models used, can be highlighted as strengths.

## 5. Conclusions

In the IBERICAN study, we find better frequencies of the components of the MetS in patients without CVD or DM and with a high level of PA, both in men and women. In addition, women present a better metabolic profile of MetS because they have higher levels of HDL cholesterol and lower levels of triglycerides, fasting glucose, SBP, DBP, and waist circumference, regardless of their level of PA and stratifying by PA. Therefore, the promotion of PA needs to be approached differently in men and in women.

We need to educate and prescribe people to practice regular PA as early as possible to prevent the risk of developing MetS and CVD, although more studies are needed to see which type of PA may have the greatest benefits. PA is one of the best investments in public health for the prevention of CVD, although a gender approach is necessary with special attention needed in promoting PA among women, who are more sedentary.

## Supporting information

S1 Fig Flow chart of patients included in the study. (DOCX)
