# Supplementary material for: Physical activity and metabolic syndrome in primary care patients in Spain
Source: PLoS One. 2025 Jan 24;20(1):e0317593. doi: 10.1371/journal.pone.0317593 (PMC11759396; doi:10.1371/journal.pone.0317593)
Supplement: S1 Fig — (DOCX) [file pone.0317593.s001.docx]

S1 Fig. Flow chart of patients included in the study
